# Book Reviews

**Published:** 1820-07

**Authors:** 


### CRITICAL ANALYSIS

OF

### RECENT PUBLICATIONS, IN THE DIFFERENT BRANCHES OF MEDICINE AND SURGERY.

---

"I would have men know, that, though I reprehend the easie passing over of the causes of things, "by ascribing them to secret and hidden vertues and properties; (for this hath arrested and laid "asleepe all true enquiry and indications;) yet I doe not understand but that, in the practical "part of knowledge, much will be left to experience and probation, whereunto indication cannot "so fully reach: and this not only in *specie*, but in *individuo*. Yet it was well said, *Vere scire* "*esse per causas scire*."—BACON.

---

*General Indications, which relate to the Laws of the Organic Life.* By DANIEL PRING, Member of the Royal College of Surgeons of London. 8vo. pp. 342. Callow, London. 1819.

FOR nearly two centuries past, the praise of experimental enquiry in the cultivation of the sciences has been a common-place theme; and it must be allowed, that the advancement of physical knowledge during that period, has been chiefly owing to the great extent to which researches of this kind have been pursued. There seems, however, to be now reason for apprehension that the progress of science is as much retarded by the want of sufficient reasoning on known facts, as it was in more ancient times by the comparative neglect of observation, and an undue bias for purely mental speculation. It is much easier to observe sensible phenomena and to make experiments, than it is to reason well on what we thus observe; and hence has arisen much of the fanatical admiration for the prevalent notions on this subject: for it is by such proceedings that many men have been enabled to make a figure amongst mankind, that otherwise would never have emerged from utter obscurity. The authority of BACON has been commonly adduced in support of those notions; but it is probable that, had Bacon lived in the present era, he would have considerably modified his precepts for the advancement of knowledge, and have attributed more importance to those efforts directed towards the generalization of the facts which have been collected since his time, and which are comparatively useless, because they have not been sufficiently reflected on. But the error which has caused the greatest waste of human industry, is that which has supposed that theoretical principles are not necessary in order to prosecute experiments with the probability of useful results; and, because some important discoveries have been arrived at by experiments made at hazard, a multitude of men have entered blindly on such enquiries, who have been unable to reason correctly even on what they may have observed. Hence the host of useless experiments, trivial observations, false inferences, loose analogies, and opinions without arguments for them, which encumber the modern records of science. The work before us is written more according to the manner of philosophizing in greatest estimation amongst the ancients: it is purely speculative. But the author does not mean to depreciate the utility of experimental enquiry: his object is to raise the former mode of philosophizing to its proper sphere, at the same time that he indicates new routes by which the latter enquiries may be extended with a probability of arriving at precise and important acquisitions in physiological knowledge.

Before we enter into a particular examination of this production, we solicit the most earnest attention of the reader; for we have to consider dissertations comprising reasonings of no ordinary depth, with original views which have been developed only by the most acute penetration; and we promise to make a display of what will amply reward the attention we require.

The object of this work is an analysis of those minute relations in the organic life of man, which result from the union of the properties appertaining to three departments,—those of mechanism and of chemistry, with those which distinguish the living from the dead state. The phenomena which are to be assigned to the properties of each of those departments, respectively, are investigated; and arrangements and classifications of them are attempted, as far as analytical enquiry has succeeded in developing the subject. After having considered in this manner the knowledge already acquired, the author states the routes which inferential reasoning appears to indicate as best qualified to lead to further important discoveries.

Here a new region lay open before him, and, on entering it, he appears to feel that he is travelling over untried ground; and therefore he professes to give on the several topics of his subject *indications* only, which do not assume the character of perfect or positive information, but aspire rather to suggest the proceedings by which such information is to be attained in the progress of research.

The work being, then, both of an analytical and inferential nature, the author has thought it right to commence with his peculiar doctrines of *truth* and *causation;* as he thus gives a precise view of the principles which regulate his subsequent reasonings, and the bases on which his propositions are logically founded. These doctrines, with the application of them to the *universal scheme of nature*, occupy the first of the three books into which the work is divided.

The first chapter commences with an enquiry into the general nature of truth; and the author, by his reasonings and illustrations, attempts to prove that belief and truth are synonymous.

We gain the belief of an existence, he observes, either by the immediate operation of an external object upon our senses, or by a process of our own minds. An external, in relation with our faculties, produces consciousness, or a belief of the existence of such external; in other words, *it is true:* and no other testimony can be cited for the existence of an external, than a *consciousness or conviction* of the reality of the existence of such external. Thus, if it be asked *why it is true* that the sun or a tree exists, the answer is, "*because I see it:*" which means, that I am conscious of its existence, or I believe that it exists. But it is found that the same object may produce a different consciousness, or conviction, in different individuals; as, if a sense be disordered, or the brain disturbed, real objects might assume extraordinary shapes, colours, and characters; or spectra might be perceived in an atmosphere which otherwise appears unoccupied. In such cases, the conviction, or *consciousness,* is as unequivocal as on points of belief which are universally received; yet the existence of these distorted objects, or of these spectra, is not regarded as true. A madman, too, has just the same testimony for the reality of his perceptions as one whose understanding is sound, namely, belief, or conviction.

The same object, then, will produce a different consciousness in the same person at different times, as well as in different persons, by operating on a different state of the faculties. Here the evidence, as far as it respects truth or reality, is the same in every case. All consciousness, then, and therefore all truth, is relative. Truth, or conviction, is identified by the relation which the properties of an external have with certain faculties: if the external be modified, the conviction with respect to it is modified; if the faculties of perception are modified, the external remaining the same, the conviction with respect to it also suffers a corresponding modification.

It is however agreed amongst mankind, that the only consciousness which shall be admitted as truth, is that which is produced by the operation of external objects upon such a state or predisposition of the senses as is *general,* though not *universal.*

Hence the author arrives at a division of truths into those which are natural, and those which may be said to be artificial: the former are, in every instance, synonymous with belief, because there belongs to them only one common testimony; that is, consciousness, belief, or conviction. The latter are agreeable with an artificial standard, which is founded on the consent of mankind; and, according to this standard, the convictions of an impaired sense or of a disturbed understanding are rejected, not because, in nature, these convictions do not equally establish truth, but because, from differences of constitution, they are not agreeable with the convictions which are entertained by the greatest part of mankind.

The author is then led to consider the nature of evidence, the grades of which are conformable with the degrees of belief which the different kinds of evidence produce. Evidence may be divided into two distinct species, *perceptive* and *inferential*: the former is experience, or results directly from an impression upon the senses; the latter does not arise from a present impression upon the senses, but is founded on analogy: thus, from a perceptible similitude in some respects, we infer a similitude in all, from our previous experience that those objects of the inference which are not seen, are in connexion with those which are seen, and the inference is justified in proportion to our experience of the frequency of the connexion.

Belief, produced by an impression on the senses, is but rarely superseded by a different belief with respect to the same object, and differs in this respect from the belief acquired by a process of the mind, from the relations of the senses with the external world being more uniform, and less complicated, than those of the understanding. But, in sensible matters, we are apt to believe more than the senses inform us of; and, in consequence of such belief passing under the authority of *sensible evidence*, the infallibility of the senses has been brought into question. Thus, a man who for the first time should see a shadowy representation of men on horseback, (like those produced by the *magic lantern*,) would believe them (his judgment not being otherwise instructed) to be men and horses of flesh and blood; and he would fancy that he believed no more than what his senses informed him of.

The author obviates such objections to his general principles as those above proposed, and illustrates his doctrines by various convincing arguments. He then gives a summary of the principles of the different grades of evidence, according to the force with which they produce either unequivocal conviction, or belief mixed with doubt.

"1st. Perceptive evidence.

"2d. Proof; or that founded on analogy, to which we know of no exception. These two are almost equal in their degree, and generally produce unequivocal belief: the

"3d, may therefore be called probable evidence; as when the analogy is rarely excepted again: thus designating this class of inferential evidence by the term which has been employed to designate the whole species.

"4th. Indicative evidence; as when the association inferred is more frequent from the absence of such association.

"5th. The evidence of possibility; when the connexion is known sometimes to occur.

"These three last, which may be considered as different grades of presumptive evidence, give rise to the diversities of opinion; for, as facts are pre-supposed to be in opposition, so their comparison will be attended with a different result, according as one or the other set of facts is recollected."

We now arrive at the chapter on *causation*, in which the author develops those principles concerning the relations of causes and effects, the application of which is subsequently continued through every section and page of the work.

He commences by a demonstration of the well-known axiom, *ex nihilo nihil fit;* and he deduces the truth of this axiom in conformity with the rules of reasoning which are previously laid down. Having assumed that this axiom is proved, the first inference which he makes to follow from it is, that there are no elementary substances or properties, or that every possible form of existence must necessarily be compounded of other forms; for, if a cause could produce an effect which is different from itself, that in which the difference consists, if superadded to the cause, must originate from non-entity; which is contrary to the axiom *ex nihilo*, &c.: if the effect be only a part of a cause, some properties having been abstracted, then there is no act of production; for that only remains, and is the effect, which was before produced. Thus, he states, "a single cause is no agent: it is a form of existence, but is capable of no transaction; for a thing cannot supply or confer that which it does not possess: it can supply only itself, or its own identity." The manner, therefore, in which a cause produces an effect, is described as involving no mystery: a cause is itself, and no more than itself, and it can do no more than exist: it may exist separately as an effect, depending only upon its own constituents; but when it, in turn, becomes a cause, and produces an effect, it is by combining with something else, and the effect is merely the union of the different forms of existence which are called causes. Thus, an effect is identified with all its causes: *it is the existence of its causes;* and the mode of the dependance of effects upon their causes is illustrated by numerous combinations, as two and two make four, because the united existence of two and two is the existence of four; and an acid and an alkali are the causes of a neutral salt, or produce a neutral salt, for the reason that a neutral salt is the united existence of an acid and an alkali; and an effect appears different from its causes, because two forms of existence, when combined, have a different perceptive relation with our faculties from that which is entertained by the same forms of existence in their separate states. The author illustrates these doctrines by adducing numerous examples to which they apply; but we must pass over them, for our limits will not permit us to enter into any particulars on this subject. He encounters all the objections that might be adduced to his doctrine, at least, we can imagine none that he has not anticipated; enters fully into the consideration of them; and, finally, shews that the alledged difficulties are perfectly in agreement with his views.

The efficiency of those which are termed *remote causes*, are afterwards treated of, and some difficulties belonging to them reconciled with the previous conclusions. The difficulty which, on first sight, appears the greatest, results from our not distinguishing, in what are considered as causes, those qualities which really act as causes, from those which have no relation to the effect. We must enter into some particulars on this point, in order to elucidate it; and we take an example given by the author for the purpose: If a steeple should fall in consequence of being struck by lightning, and a man, passing by at the time, should be killed by a stone falling on his head, it might be said, would not the lightning be the cause of the death of the man? and, if so, how is it that the lightning, according to the author's doctrine, converts a living principle into the condition of death? Did the lightning exist in the stone, pass from it into the man's brain when it fractured his skull, and mingle with the living principle, changing its identity from the living to the dead state? This difficulty the author thus explains: As we find that death may arise from a somewhat similar accident where there is no lightning in the case, we must conclude, that the lightning does not in this instance mingle with the living principle, and that it is a cause of death only in respect to concurrent agents, with which it is so related as to produce an effect conjointly with them. Allowing, then, that the stone contained lightning at the time that it fractured the man's skull, the relation between the agents might be, that the bone received properties only common to an impulse of any kind, and had no relation with the lightning by which it might be derived to itself from the stone; or, supposing that the bone participated in the lightning, yet the effect on the vital principle would be caused by other parts of the agency, and in no degree by the lightning, provided the relation of the latter with the series ceased with its passing into the bone.

The foregoing is an example of a gross and simple character; but it will prepare the reader for the following one, which is exceedingly happy, and must render clearly intelligible this particular point of the author's doctrine, as well as elucidate his proposition, that *the cause always exists in the effect.*

"A man made a watch: the man is the cause, as may be said; the watch the effect. But surely the man is not included in the watch? Why, no: truly the man has an habitation in England, and the watch may be lying upon the dressing-table of a gentleman at Calcutta.

Here we must trace a process of causation; and, though an example more palpably opposed to our doctrine will not readily be devised, we shall still find that our principle is untouched by it.

"The vital organs of the man, his animal powers, his mechanic knowledge, his facility in the art, all concur to produce one effect, which is the exertion of that ability which is produced by this complicated causation; this effect, in its turn, becomes a cause, by which the parts of the watch are adapted to each other; the intelligence of the artificer is related with volition, or produces and modifies volition; volition is related with the muscles of the arms and fingers; and modified motion, (motion modified according to the volition,) is the result; by this motion the parts of the watch are prepared and adapted. So that it is not a man with which a watch holds a relation as with a cause, but with a certain moving power, which is the first cause, proceeding from the man, which is exerted upon the works of the watch. If we would know whether this power of motion, or its properties, communicated to the works of the watch, still exist in them, we can scarcely answer this question, without a better understanding of the relations of moving powers in general. The power of motion appears to be expended in the act to which it gives rise. And, whether it enters into the substance moved, or whether it is communicated from the subject moved to the surrounding medium, in the course of its progression, is a point which in this place it is superfluous to discuss. The watch being thus produced, is then identified as an effect by its own constituents, and is maintained by relations subsisting between its parts. The powers which concurred to produce it are its remote causes, and these may be withdrawn, or cease, while the watch preserves its identity: its real, true, or efficient causes, are those by which its identity is preserved, when its connexion with the remote or concurring agents has ceased; and these causes cannot well have a place in England, while the watch is at Calcutta. All this is very obvious, and requires no more to be said about it."

Many apparent difficulties arise from *succession* of phenomena being confounded with cause and effect; all which the author takes into consideration, and he endeavours to give to the evidence of succession its true distinctions and importance. The whole object of this chapter, as far as it respects science, is, by defining the laws and modes of causation, to conduct enquirers to deeper objects of investigation than have usually occupied their regards, and to inculcate a sort of analytical enquiry, which is required for a satisfactory understanding even of the most simple subjects.

The third chapter is an application of the preceding doctrines of causation to the general phenomena of nature. This chapter is entitled "Universal Scheme in connection with the foregoing Principles." According to these principles, the author examines the subject of creation, or origin of forms, and treats copiously of the government by which natural processes are directed, and of the harmonies and discords which are observed or assigned, and whence arguments have been deduced of an intelligent principle working amidst physical agencies, and directing them to their final purposes. The author's views are here peculiar, and the result of his investigations does not favour the proposition of a theology upon natural proofs; at the same time he admits the testimony upon which the Christian theology is proposed, and shows that, in conformity with his rules of reasoning, propositions may be accepted, or facts believed upon authority, or by faith in the veracity and competency of witnesses; of which propositions, or of which facts, we have no physical testimonies. Creation, according to natural evidence, the author considers, appears to express only the period at which certain changes occurred in the forms of things which previously existed, but which were otherwise combined. This conclusion is deduced from the principles, that a cause cannot supply that which it does not possess, and consequently *that* in which the effect differs from a single cause, must have arisen out of nothing, unless supplied by other causes. Hence it appears that nothing can be produced, the constituents or causes of which did not before exist; that every apparent origin of being, is only a new combination among previous forms of being; and that, as every cause must supply its own existence to the effect, and as nothing can happen without a cause, so *nothing that is* can be annihilated; for, as all causes *must produce existence*, so none can produce annihilation.

A scheme of necessary concurrences among physical agents, is then exhibited; and a necessary harmony is said to result from the causation which is perpetually going on, "since, if there were not this harmony of relations," says the author, "such a state of things could not subsist: if there is not agreement and compatibility in one state of things, things will be compelled to adopt another, in which there is agreement and compatibility."

A rapid sketch of the order of causation, and of phenomena, on physical views, with the confession of a scheme founded upon authority, which is capable, in some points of disagreement, of superseding the conclusions which are founded upon natural proofs, fill up the remainder of this chapter.

The physiological disquisition commences with the second book, which is entitled "On the Organic Origin of Man." The author considers the division of the constitution of man into the organic, animal, and intellectual, departments, as indispensably convenient, though in some respects imperfect, and conformable with the division of which nature has on other occasions furnished us the examples: he consequently adopts it in his discussions, though, he remarks, they are perhaps not so distinct as they appear, and it is possible to regard them all only as modifications of the first. He then treats of the origin of man by *derivation* from beings of indentic nature, and commences with some considerations on the formation of the maternal ovum, and the history of its properties, previous to their assemblage in a sensible rudiment, which necessarily lead him into a discussion on the origin of vitality. The object of his arguments on this point is, to show that organic life depends on the presence of some principle not in any way evident to the senses, as death takes place without any apparent alteration of the chemical or mechanical alliances of the body; and that this principle not only preserves the life of the body, but also produces the union of the elements of the textures. This principle he terms the *organic spirit*, for the purpose of distinguishing it from sensible material and chemical alliances; but it must be remembered, that, in conformity with his doctrines of causation, this spirit itself cannot be considered as a simple elementary principle, but as containing a multitude of properties by which altogether it is constituted. Those doctrines teach us, also, to expect the agency of properties in phenomena, where we are not even acquainted with their effects: the existence of bodies compounded of bodies related with the senses; these latter, of others not related with the senses; and thus we are led to trace the relations between a visible and an invisible world.

The first sensible origin of man is his existence in the ovum, the history of which the author now takes into consideration. The inferences respecting the origin of which that appear most probable, he thinks, are, that the ovum of the female, and the semen of the male, contain vital properties which are derived from every seat of the parents, and that thus the ovum becomes the similitude of the progenitors, requiring only a progressive causation amongst its inherent properties, with the aid of a material nutrition, in order to establish resembling functions, and to be developed into a similar organization. Properties corresponding with those in every seat of the mother are supposed to be possessed by the maternal ovum; but this is a state of informal life, and the properties of life in this stage are said to be disposed for quiescence, owing to their forms of combination. The seminal influence holds such a relation with these properties, as, from being quiescent, to render them active: the quiescent state of combination is then dissolved, and progressive causation among inherent properties established, by which, through many grades and approximations, they finally assume the character of formal and independent life. Properties of life combine and assume their spheres, in agreement with their peculiar laws; and they, in every sphere, develop corresponding textures, by an affinity with certain constituents, which they withdraw from a common material of nutrition. A gross illustration of this agency may be given in a fact observed in the vegetable world,—that the slip of one tree, as a peach, may be grafted on a tree of a different species, as a plum, and yet produce its proper fruit, though its nutrient materials must be precisely the same as those of the stock to which it is fixed. The series of phenomena which occur in the formation and growth of the ovum, are minutely traced in their supposed historical order; and the author here professes the same object as that which the whole work entertains, namely, to exhibit an inferential system, which might serve to aid and extend the researches and acquisitions of experimental enquirers. All the difficulties which occur are fully considered, and explained by the principles of causation where these are adequate to such explanation; and, when any one inference is not positively justified by the evidence, he states the alternatives which are to be settled in the progress of research.

The origin of man by *constitution*, that is, by original creation, which may be supposed to be previous to that by derivation, is considered in the third chapter.

This chapter is merely an application of those laws of causation, and of origin of forms, which are before said to be universal, to one particular example. It is stated as an examination merely of natural testimonies, an experiment to try how far, or to what conclusions, unaided reason is disposed to carry us, without designing to infringe the credit of accounts upon this subject which are proposed upon higher authority. The difficulty of attaining any precise knowledge of the period or place of the origin of the human species, is stated and discussed, chiefly with reference to traditional and recorded evidence, and the universality of a deluge. The difficulties attending the consideration of the origin of man above alluded to are extreme; but the author pursues them to their utmost extent, with the peculiar depth and acuteness of reasoning which characterize his previous disquisitions. Our limits will not permit us even to designate the principal of his several propositions; but, in reference to that of the constitution of man by many approximations of his constituents, we may observe, that the author states that the *sources* of life are in the earth, the air, and the waters. The elements exist in these *uniformally*; it requires only changes of combinations to give to these constituents their distinct forms, in order that they should assume the character, and produce the phenomena, of life. And how then, he says, do changes of combinations arise in this or any other department, but by the force of relations subsisting among constituents? Adequate causes must be provided for every effect, and the effects which do take place are those of which these are the causes. Every form of life is only a predisposition to another: death itself, which is only a modification of life, becomes a source of life, alternately furnishing elementary properties to the higher, the lower, and the more complicated forms; and then mingling with, and assisting in, the formation of those belonging to the most simple and the lowest classes. It is probable, then, he thinks, that, at a remote period of the world, the constituents in it from which the organic spirit of man would result as an effect, were disposed to unite and produce, as a separate combination in nature, the spirit in question. The concurrence of constituents at this period must have been determined by one of two modes of causation, or by both, namely, the constituents previously disguised in another constitution, were suffered to form the identical spirit by an agency which detached them from their former alliances; or by an agency which furnished additional properties to constituents which were not otherwise identical. These agree with the only possible modes of causation, by subtraction or by addition of properties, or both.

We now arrive at the third book, which treats of "Post-fetal Life," and is divided into five sections; the first of which commences with some considerations, of a general nature, on the "condition of the spirit,"—a term which the author adopts to express those properties whose existence is inferred from their effects, and which are thus contra-distinguished to the visible structures and products. The existence of this principle, that is to say, the above properties collectively, being inferred from its effects, the next object is to class those effects, and give corresponding denominations to the properties of the principle by which these effects are accomplished. But, as the author makes it appear, the progress in this analysis and classification is not to be boasted of: "the most that has been done," he says, "is to designate three or four species of contractility, from which has originated a vast deal of erroneous and absurd reasoning;" and he shows the inadequacy of all the doctrines founded on the degrees of excitement, contractility, &c. to explain the phenomena in which properties of life are engaged. But, as we shall hereafter have occasion to return to those several points in a particular manner, we pass on now to the next chapter, which is on "the mode in which life is maintained." This comprises a multitude of considerations of the most interesting kind.

The author, by his arguments and illustrations, shows that life is not to be conferred on the body by the only externals which support it, namely, by food and by air; but that life itself, that is, the organic spirit, must operate upon air and food to the end of its own perpetuation. The following paragraph comprises the sum of his doctrine on this point:

"The principle of life, or, as we have hitherto expressed it, the organic spirit, exists in every part of the textures. Blood, containing the elements of life, which are furnished by its two sources before named, viz. air and food, is everywhere diffused among the textures. The exposure of that which contains the elements of life, to life itself, is in this way complete. The next operation is simply this,—that life, by an affinity subsisting between itself and its elements, separates them from a common material, and unites them."

This act is described as being perpetual: life is no sooner formed than it vanishes, or changes its form, or dies; and the principle would become extinct, but that it re-produces its resemblance in uninterrupted succession from its elements, which are contained in arterial blood; which fluid is, in the words of the author, "*a further preparation of the informal life which exists in earth and air.*"

Thus, there is no permanent *quantum* of life, but it produces itself from its elements, as fire does itself from combustible substances, and lasts no longer than it is capable of perpetuating its resemblance by its affinity with successive quantities of its elements, which are derived from earth and air. All the properties of life, those which are passive, as well as those which are active, are renewed in this way; and this is described as the mode of causation by which assimilation takes place. Thus, every animal and every plant maintains in perpetuity its own characteristics; and thus every body preserves, for the future, a conformity or resemblance with the past; all obeying the common relation of cause and effect, according to the author's doctrine of causation, which is here admirably exemplified, at the same time that it is illustrated, existence forcing existence identified by the properties of its causes.

The above must, however, be considered only as a general view of the laws of life in this respect. The preservation of a species is interrupted by various accidents, and the perpetuation of the past identity of an individual is interrupted, "by the development of latent causes, which are internal, and by varieties in the nature and operation of externals." It is by the former law that the organic changes take place which are conspicuous in the several stages of life; and by it the order of disease is, for the most part, regulated. The origin and operation of these means are fully discussed, agreeably with the author's doctrine of causation and those relating to the origin of the ovum; and then the influence of externals, in relation to the second law above indicated, are taken into consideration. The numerous indications the author presents are of the highest importance; but our limits will not permit us even to designate them, much less to attempt their development.

The next chapter treats of "growth." The phenomena of growth are chiefly considered with reference to the process and laws of the assimilation of the organic spirit. The principle of life, or certain properties of it, existing in every minute sphere of the organized fabric, is described as first holding a relation with its own elements, by which life itself is constantly renovated, and the elements or constituents of the principle are conjectured to hold an affinity with solid particles in the nutrient material. Every modification of life has its relation with certain or peculiar organic particles; hence, according to the life of respective spheres, will be the character of the structure with which it is inherent. Life is thus said to form itself from its elements; and in this process of assimilation, by which properties of life are drawn from a common material, organic particles are also separated, with which elementary life was in previous alliance. This subject involves many difficulties, which the author states; and, as known facts are not equal to a settlement of them, he presumes on the accuracy of his inferences no further than is warranted by the evidence, and, indicating the line of enquiry, leaves the decision to the future increase of facts. The development of the structures is also considered with relation to the quantum of life; and here we find, as indeed we do in every page, a multitude of indications of the most interesting and important nature, and especially, in this instance, in relation to the origin and development of disease. The following are some of the author's conclusions on this subject, which are here adduced because they are of a general character.

"1. The quantum of the spirit depends upon the quantum of the elements exposed to it, which are contained in the blood.

"2. The quantum of the elements depends upon the preparatory functions.

"3. The disposition of the spirit, in regard to the structures, is regulated by latent causes which belong to it.

"4. The quantum of the textures depends, 1st, upon the quantum of the disposed spirit, and, 2nd, upon the quantum of the organic particles in the material.

"5. The quantum of the organic particles in the material, depends also upon the function of the preparatory organs."

Another point of view in which the subject of growth is discussed, is with respect to the absorbent function. Here we must fairly confess our inability to give any thing like even hints of the author's propositions, they are so multitudinous, and all so intimately connected; at the same time that we acknowledge the very great degree of interest attached to his numerous original indications. The whole of this chapter bears very close indeed on the practice of medicine; but, as we shall hereafter find others that still more intimately relate to it, we must proceed to the next in order, which is on "animal heat."

The discussion on this subject, too, will not admit of abridgment; but we may state, that, after showing that there can be no source of heat in any one organ or structure, he thus explains the formation of animal heat. It is proved, by the influence of heat in incubation, that it is necessary to identify the living principle of such animals as possess it; it continues in animals as long as life lasts, and helps to constitute, or is a part of, the principle of life. This principle renews itself by assimilation from arterial blood, which contains all the properties of life, and those of heat in an informal or elementary state. Thus, as a principle assimilates, or renovates itself from its elements, heat, as a part of that principle, is, in the same way, and at the same time, produced. The arguments for this doctrine, together with the objections or difficulties which suggest themselves with respect to it, are stated and discussed.

In the fifth chapter the author analyzes the relations subsisting between vital, chemical, and mechanical agents. The relations of these departments with respect to each other are traced; and it is attempted to assign the respective shares, influences, and co-operations of the properties belonging to these classes in the phenomena in which they are mutually engaged, and which comprehend every possible process or agency which can occur in an animal body.

In the next chapter, the relations of the properties of life with each other in different seats are considered. Many points of abstruse research are here entered into in the analytical way; an application of the doctrines of causation being here made, and the investigation conducted upon the views which these doctrines suggest.

The evidence which is afforded by the ordinary methods of experimenting, and particularly upon the nervous system, is minutely scrutinized and defined; and exposition made of the fallacious reasonings by which conclusions in physiology have been deduced from experiments made without any precise views, and conducted in a loose manner.

The relation of vital properties in one with those in another seat, are stated to be, 1st, direct; and, 2d, indirect: direct, as when, the properties being affected in one seat, the influence of this affection is communicated to those in another seat, without any change in the alliances of the latter, whether chemical or mechanical; indirect, as when the function of an organ whose office it is to prepare the chemical for the use of the vital properties in other seats, becomes impaired, in consequence of which those properties are elsewhere affected, by a disturbance of the relation which subsists between them and the chemical or other products of the organ, and not with vital properties belonging to the organ. The more evident examples of the latter are furnished in the preparatory organs, as the stomach, &c. The above relations, as well as the properties of life belonging to particular seats, are finally arranged under the following classes: 1st. Assimilating properties; 2d, regular dependant, and, 3d, occasional properties of life. The first is the organic life of every seat which renews itself by assimilation, and is dependant for its renewal only upon its own existence, and the supply of arterial blood. The second class (the regular dependant properties of a seat), refers generally to that functional life of organs which is identified by receiving the properties of a distant seat. This class of properties is indicated, by experiment, in some functions of organic life, as in those of some of the secretions, and is illustrated with less ambiguity in the dependance which the muscles of respiration acknowledge on a communication with a centre of nerves. The third class (or the occasional vital properties of a seat), includes all those instances in which properties of one seat are made, by an occasional cause, to influence those of another, between which there was no habitual dependance, no reciprocation of function; as when the action of the heart, or the function of the brain, is disturbed by infliction of an injury in an extremity, or by a disturbance of the natural relations and spheres of spiritual properties. This class relates chiefly to disease or preternatural condition. The two last classes do not maintain themselves by assimilation in the secondary, or communicated, seats; for they would live independantly of a communication with their sources, acknowledging, like the first, a dependance only upon arterial blood. This division is a leading one throughout the subsequent sections. It is not right to praise any particular part of this work; but we have deeply felt this restriction on the expression of our sentiments at the conclusion of the consideration of this chapter, as well as of several others that we have already passed. The chief design of the chapter we are taking leave of appears to be, to inculcate that strict analytical enquiry which the author has sketched in his preliminary disquisitions, and to show the applicability of his principles to the general objects of scientific investigation.

The second section of this book treats of the preparatory organs of the nutrient materials; and the first chapter contains a more minute or extended view of the previously considered relations of life, and of the modes of enquiry which are adapted to their elucidation. From general views the author descends to more particular investigations; and the second chapter, on the stomach, commences his indications for an analysis of particular functions. The following is the leading divisions of the topics of this chapter. The relation subsisting between food and the function of the stomach is to be considered (conformably with the general division before expressed),

"1st. According to the mechanical relation subsisting between food and the structure of the stomach.

"2nd. According to the relations between the chymical constituents of food and those supplied by the stomach.

"3d. According to the relations between the vital properties of the stomach and the properties of the same kind in food: thus far they may be considered separately. They are also to be considered reciprocally; that is, as the stomach acts upon food, and the converse. They are also to be considered as regular and occasional. Lastly, their mutual or conjoint agencies are to be considered according to the following order:

"1. *Mechanical relation.*
"2. *Chemical relation.*
"3. *Spiritual relation.*"

Each of which is subdivided, and the ends of the specification fully exposed. We must again state our inability to give any thing like a satisfactory abridgment of this, and of the ensuing chapters on the preparatory organs; for every paragraph, unless employed in the examination of received opinions, contains a topic of research, or an indication for additional enquiry. The order of investigation which the author adopts is everywhere of the analytical kind, and every inference gives rise to an original indication. We have not much space left for this article, and we are desirous to arrive at the part of the work on the nature and origin of Disease, on Therapeutics, and on Death.

The third section treats of the relations of Blood, and its products, in its vessels, and in the several places of its distribution. The chapters of this section are enumerated under the titles of "the Formation of Blood," "the Lungs," "Arterial Blood," "Relation of Blood with the Heart," "other Relations of Blood," "the Absorbents," "Secretion," and "Relations of the Organic Life in the Nervous System." These chapters contain few or no theories, and consequently there are no doctrines to be exhibited. The enquiry is, throughout, an indication for further analyses, and the development of the subject is conducted upon rules of the closest reasoning. The object of the author is here, as in the other instances, the discovery of *efficient causes*; and his reasonings and indications with a view to the future attainment of this description of knowledge, are applied to the most abstruse and nearly inscrutable processes.

In the last chapter of this section, on the relations of the organic life in the nervous system, the relations of the properties of life in this system are traced in the analytical way, conformably with the division of the properties of life into three classes, which has been already quoted. The chapter is occupied in considering the several relations which involve the properties respectively, and mutually, of either class. The rules of conducting experiments with a view to ascertain the relations of the nervous system are sought after and defined, and the kind and degrees of evidence specified which belong to the facts from which relations are inferred. Among other relations, that of electricity with the properties of life is considered. The author regards all the attempts to identify life and electricity as little better than puerile: he considers electricity as intimately connected with life, and capable of influencing, modifying, and even substituting, some of its properties; but, after enumerating a multitude of particulars in which life and electricity are totally different, he concludes, that the phenomena of electricity furnish no general analogy by which their identity with life can be correctly inferred.

The fourth section is on the general nature of Disease; and, in the first chapter, the author makes such an inferential analysis of disease, with a view of tracing its origin, as is conformable with his doctrines of causation. He begins with a definition of health, predisposition, and disease, and states such examples of them as serve as a point of reference to the subsequent discussions into which he enters. He distinguishes disease into primary, secondary, and general; and its origin into accidental or spontaneous.

"Primary, as when it originates in one seat, by a causation peculiar to the properties of this seat; secondary, as when disease is extended from the original to a related seat; general, as when, in some form, few parts of the system are exempt from it; accidental or foreign, as when disease is produced by external agents, which are related with properties common to the healthy condition of the species, as in the instances of wounds, poisons, &c.; spontaneous, as when disease happens without any external assignable cause; or as when it is excited by a natural cause, as air, food, &c. operating upon a state of constitution which does not belong to the healthy condition of the species."

The author traces the origin of disease to a spiritual predisposition, or a predisposition made by latent properties of life; and the development of disease is supposed to engage a series of changes in spiritual properties, which is commensurate with the first formation of the ovum. The manner of progressive change among constituents, by which passive spiritual properties become active, by which disease occurs, and by which all spontaneous successions of phenomena, and all the conversions of the structures are determined, is thus described:

"Progressive change is accomplished by reiterated causation. If a thing preserve an uniform identity, it does so because it is surrounded by no agents which are so related with as to affect it; if a thing is once changed, and then preserve its new form, it is that it is exposed to the operation of a related agent, and that the form which it assumes in consequence ceases to be related with the existences which surround it; if a thing suffers one change, and then another, and a third, through a lengthened series, it is because each successive form of existence has a causative relation with other forms."

The application of this doctrine of progressive change is frequently made, in aid of the subsequent investigations, and its application to the sensible properties of the body is familiarized, at the same time that it is illustrated, by a rapid sketch of the development of the moral properties. Here, as in the rest of this section, the author discusses the systems and opinions relative to this subject which are generally received, when he develops his own peculiar views, always connecting with his doctrines the evidences by which they are supported.

If any part of this chapter may be considered more estimable than the rest, it is that which relates to predisposition to disease: it is an admirable example of the utility of the author's doctrine of causation in pathological reasoning, and merits the deepest meditation.

The indications we have already met with on the subject of disease, and the illustrations with which they are accompanied, disclose a multitude of things of the deepest interest and importance to the physician; but, as the pursuit of each series of them might furnish matter for the labours of a man's life, it must be long before the good that may be expected to emanate from this work will be realized; and we venture to prophesy, that, in this long interval, many men of mean talents will draw secretly from it, and sport their little lights, to glitter for a time amongst mankind, whilst they endeavour to obscure the name of Daniel Pring and his "Indications," with as much anxious care as shrewd and eminent churchmen have used to conceal the "Court of the Gentiles" of Theophilus Gale.

In the second chapter the author considers the more precise origin of disease in one seat, agreeably with the doctrines of progressive causation above expressed. This chapter is little more than an illustration of principles before laid down, and the author thus states the axioms he means to establish from the illustrations just adverted to.

"1st. That every primary spontaneous disease is produced by progressive change in the constituents of its seat.

"2d. That this progression may be interrupted, when the present state ceases to find a causative relation with existing causes.

"3d. That, if the progression of change is resumed, it is because new causes obtain a relation which did not before exist with the constituents of its seat.

"4th. That these new causes may come to produce change in a given seat, either from progressive internal change among connected properties, or from exposure to an external cause which is related with the present predisposition of the seat; these might be complicated."

The difficulties in tracing the history of spontaneous disease, although the laws relating to it are so few, are by no means trifling: we take an example from amongst those the author has adduced as the subject of discussion.

"Say a tubercle forms in the lungs: why does it form there? The part, it may be said, becomes thickened by coagulable lymph, which then becomes organized, grows, suppurates imperfectly, &c. Why was the lymph thrown out? From inflammation. Why did the inflammation occur? Excited by cold. How came the part predisposed to such a relation with cold? It has somehow attained such a state. Now my abstract refers to this word *somehow;* and, if this somehow is to be answered, the only reply that can be given will be found in the above propositions, which I have called axioms. To leave, then, this subject of the manner in which disease begins, and without taking any farther with us the incumbrance of these views, we will simply say, when disease occurs spontaneously without any assignable external cause, that it happens from the development or operation of latent causes, about which we have been of late so busy; and, that when disease happens from an external assignable cause which does not produce the same effect in others, or in the same individual at other times, we will say that a predisposition existed to the operation of such cause, of the nature of which predisposition also enough has been said in the way of indication."

The causes of disease, then, are of two kinds: "1st, those which, being related with the spirit (the vital properties), have the power of affecting it, and producing disease as often as they are communicated, or as long as they continue to reside with the spirit; 2d, those which may modify for a time, or permanently, the identity of the spirit, even though the operation of the primary cause should have ceased. The state of the spirit produced by the first class of causes is not an assimilating one; the state produced by the second is either maintained by assimilation, or runs into a succession of modified states, each capable of assimilation."

The rest of this chapter is occupied in discussing the relations of the chemical and mechanical, as well as the vital, properties, to the origin of disease; and, having traced the origin of disease to certain states of the organic spirit, or, in the common term, the vital properties, he considers, in the third chapter, "the general nature of the disease of the spirit," and especially the laws respecting the duration of disease. We must here also state our inability to give an abstract of the author's analytical and inferential disquisition: we can only say, that it throws a new and brilliant light on the peculiar nature, as well as on the distinction, of many acute and chronic diseases. It contains, also, some very ingenious notions respecting the causes of the insusceptibility to several diseases a second time, and the reasons why this insusceptibility is occasionally not acquired.

The fourth chapter is on "Disease of the assimilating, of the regular dependant, and of the occasional, Properties of Life." The objects of this chapter are, to show what classes of disease belong to, or interest respectively, these classes of properties. The modes of discriminating between the affections of one class and those of another are sought after, and the intercourse and re-agencies of each class are exhibited, with appropriate illustrations. The degrees of evidence which succession furnishes with respect to the relation of cause and effect, is here recapitulated with especial minuteness; and the author exposes the grounds upon which true or false inferences of causation are liable to be made from this description of evidence. This chapter cannot be too deeply meditated on by the medical practitioner; and the subsequent one, "on the general Nature of related Disease," is hardly less interesting to him.

The author divides related diseases into two classes: those in which a primary ceases upon the occurrence of a secondary disease; and those in which disease continues in a primary seat, and runs its course independantly, notwithstanding it produces consecutive diseases in other seats. The first the author designates as "a substitution of disease;" the second he denominates "related extension of disease." The author here sketches numerous examples of both classes. He considers the formation of fat as a substituted disease, and the one which, of all others, occurs the most frequently. The formation of fat, he thinks, tends to maintain health by *defining a harmless seat of disease* while it lasts, and it is seldom spontaneously removed without the occurrence of a substituted disease in some less convenient seat. He gives a rapid sketch of several examples of disease of this class, and then enters, in a similar way, into the consideration of "extension of disease." After having adduced sufficient illustrations of diseases of this class, he discusses the chief of the prevalent doctrines, and shows how little many of them will bear the test of analytical examination. He enters more particularly into the refutation of those doctrines which assign, arbitrarily, one organ as a common, or universal, seat of the origin of disease. The bad reasoning of the pathologists who maintain these views, is freely exposed; and the author concludes this chapter by a citation of facts and evidence which bear upon the subject, and shows, upon the credit of this evidence, what inferences are to be legitimately drawn. His examination of the prevalent doctrines about "metastasis" and "conversion" of disease, relates in the most intimate manner to the practice of medicine, and may therefore be designated as the part of this chapter that will be most generally interesting. The author's whole course in this chapter is strictly analytical and inferential, and we recognize at every step the powers of reasoning that produced the disquisition on causation. We could adduce from it many striking examples of the necessity of our extending our views beyond those from which the pathology generally received has been sketched, if we would obtain real knowledge of the nature of most diseases; but our closing limits oblige us to proceed to the chapter on Therapeutics.

The author commences with a retrospect of his doctrines of diseases, on which his therapeutics are founded, and then enters into the consideration of the application of remedies; and his doctrines of causation are again brought into active service on this very important occasion. We can here adduce nothing but some of his more general propositions; and first his classification of remedies, in the application and arrangement of which he afterwards proceeds. Curative remedies, he says, may be divided into three classes: "1st, those which cure by a direct relation with the cause of disease; 2d, those which cure by removing or obviating a perceptible (or sensible) cause of disease by an intermediate relation; 3d, those which cure by latent causation." These classes are respectively exemplified. The subdivision of the third class, or remedies which operate by latent causation, are thus enumerated: 1st, those that cure without a sensible operation; 2d, medicines that produce cure by sensible change, or by sensible effects; 3d, medicines that cure by employment on the seats of disease, with or without sensible effects; 4th, medicines that cure by an operation upon *related seats*, with or without sensible effects."

This subdivision is thus exemplified:

"1st. Medicines that operate without sensible effects. In this manner arsenic may cure intermittent fever, scorbutic disease, &c. In this way diseases are cured by bark, by antimony, and mercury, in small doses, &c. The cure in these instances is accomplished by successive causation, and, consequently, the relation of the remedy with the condition of the disease is mediate.

"2d. Medicines that cure, producing sensible effects or disorder of their own. The remedies which fall under this class are by far the most numerous. To this class belong all the emetic and purgative medicines, arsenic and mercury in large or frequent doses, antimony, steel, henbane, hemlock, digitalis, blisters, bleeding, issues, &c. These

Page(s) missing remedies also operate by successive or intermediate causation, without removing, in the cases we are supposing, known causes.

"3d. Medicines that cure by employment upon the seats of the disease. In this way all those remedies act which are applied to individual seats through the medium of the circulation. In this way, also, the topical remedies act; such as cupping, lotions, blisters, &c. These operate by mediate relations; and, in the cases we are supposing, that is, where the precise cause of disease is not ascertainable, by a train, or succession, of latent processes of causation.

"4th. Medicines that cure by an employment upon related seats. Thus, a disorder of the head is cured by emetics, or anasarca of the legs by purgatives; or a disorder of the head, existing beneath the cranium, by the actual cautery to the scalp; or a disease of the eye, by an issue in the arm or a seton in the back of the neck; or as a disorder of the stomach is cured by purgatives, or an inflammation of the pleura by blistering the skin; or as a disease of a joint is cured by a caustic issue, &c. These remedies act also by successive causation, or by a series of related processes."

The author then proceeds to connect the doctrines which he has formed on this subject with those pathological principles before laid down, after the following manner:

"1. Primary disease, in all instances, consists in a change, or modification, of the principle of life, compared with the state of health.

"2. Primary disease is a state of the principle of life compatible with its assimilation; and in this way it is maintained.

"3. Secondary disease may be maintained after primary disease has ceased.

"4. Secondary, or rather consecutive, disease may be maintained in two ways after the primary disease has ceased: 1st, if such is the relation between the life of different seats, the influence of the primary upon the secondary may be to produce a modified assimilating state of it, as if a swelled testicle should follow the irritation of an injection, and degenerate into schirrus, long after the urethra has resumed its state of perfect health; 2d, the consecutive disease may be maintained by an effect of the primary, which does not arise out of a direct relation between vital properties, as when primary disease changes the textures or the secretions, or produces foreign substances, &c. The consecutive disease of the spirit may then be maintained by these causes, and these causes being removed, it ceases, because the relation of their properties with the spirit is not to produce a modified assimilating state of it. These causes, or effects of primary disease, may be distinguished as the material occasional causes.

"5. But primary disease may maintain consecutive disease by direct relation of the properties of the principle; and, this secondary state continuing only so long as the primary lasts, the properties producing the secondary disease may be distinguished from those producing a modified assimilating state, as the occasional spiritual causes of disease: as when the respiratory organs are disordered by pressure upon the brain, as by bone, and resume their natural state of properties when this pressure is removed.

"6. Every disorder of the spirit which is not maintained by the occasional material causes, is maintained by assimilation: for, if the disorder should be of the secondary kind, and does not assimilate in its seat, it is dependant upon a disorder which assimilates in some other seat, the present sum or quantum of the spirit requiring to be perpetually renewed, and acknowledging no other source than that of assimilation.

"7. Remedies operate either by removing a known cause, or by latent causation. It is an object in medical investigation to discover the cause of disease; for, though every internal cause which we are capable of knowing must be a consequence of previous disease, being once formed, its existence may be independant of that primary or previous disease which produced it. In such a case, the only present condition of spiritual disease may be that dependent upon a material occasional cause, which being removed, the existing spiritual disease ceases; as when, by the operations of surgery, (to advert to palpable instances,) the material effects of previously diseased actions, as they are called, are removed, febrile or other consecutive constitutional disease, engaging properties of the spirit, and maintained by such occasional material causes, ceases.

"8. But most remedies operate by latent causation, or by successive causation. The operation of remedies may in this way acknowledge a complication equal to that which has been described of disease. The relation of remedies may be directly with unknown material occasional causes, or indirectly with such causes; or, in a few instances, directly with the state of primary disease; or, more frequently, indirectly with the state of primary disease; or with secondary assimilating disease, directly or indirectly; or, directly or indirectly, with secondary occasional disease.

"9. Those diseases are the most numerous in which no cause, as of the material occasional kind, can be assigned. We are not justified, in these instances, in inferring the existence of such cause by general analogy, or merely for the reason that diseases are sometimes known to be maintained by the material occasional causes, since disease must originate independently of such causes in every instance, and since the instances in which such causes are ascertainable are comparatively few. When therefore a cause of this sort can neither be known sensibly, nor assigned in agreement with any particular analogies, we are justified in concluding only that the modified phenomena which depend upon spiritual agency, are produced by a state of disease which consists of a modification of the healthy state of the spirit. Conformably with this rule, we should infer all diseases of function, which are not dependant upon the material occasional causes, to consist in a modification of the properties of the spirit.

"10. As all diseases of the spirit are maintained by assimilation, it remains that we should consider the agency of remedies with respect to that process by which disease is maintained."

The concluding section is on "the Death of the Organic Life;" and the author, in the first chapter, considers "death in connexion with disease." Death, he says, happens either, "first, from defect or modification of those causes which maintain life, and which have been sketched in our physiology; 2d, from the operation of certain related externals." The history of death begins with the history of disease. The origin of the spontaneous processes which terminate in death, is in the vital properties; but death may take place either, "1st, from primary, or, 2d, from secondary disease." These modes are then elaborately discussed in reference to those inferred properties of which life, according to the author's system, is compounded. The second chapter of this section is "on death produced by external causes." The modes by which death is produced by infliction of external injuries on organs, are thus arranged, either,

"1st. The spirit residing in these surfaces receives a modified supply of blood, as by the general injury, the compression, &c. which the vessels must sustain by a breach in a structure before continuous; or,

"2d. The mechanical change of the structure must directly produce a change in the condition of the spirit; or,

"3d, The spirit is directly modified by the confusion, or mixture of spiritual properties, which before held distinct spheres."

The chapter terminates with a discussion of those points; and the author then treats of the "general nature of death of the organic life." An attempt at an inferential analysis of the question, what becomes of life at the time of death? is attempted; but, without a more intimate acquaintance with the author's doctrines respecting the "organic spirit" than, we fear, our analysis has enabled any one to attain, it is hardly possible to convey to the reader an idea of the disquisition on this intricate and apparently inscrutable subject.

The author, lastly, takes into consideration the phenomena of the transfusion of life, or those by which the remains of animals furnish life to other forms of it; and concludes by remarking, that "one law appears universal in the translation of life and organic particles, viz. that, if the causes of the living state of one animal live again in another, they live, not according to their own nature, but according to that of the new animal which they help to form, and which preserves his identity under any variety of the means of nutrition, provided they are to him means of nutrition; that is, he takes out of them only himself."

Had we been able to enter into the consideration of this work in a more particular manner, it would have been superfluous to have expressed our opinions respecting its utility and merit, for it is only necessary to peruse it in order to discern them; but, as it is itself of an abstract nature, the greater portion of it will not admit of abridgment: we have therefore been obliged to give a character only of many chapters and sections, instead of an analysis of them. It becomes proper, then, for us to state, that we think it qualified to produce inestimable benefit to medical science. We have perused it several times, and, the more we meditate on it, the more earnestly we are disposed to regard it, not merely as an honour to medical literature, or even to the age in which it has appeared, but as one of the greatest productions of the human mind.

In a work of so extensive a scope as that which this comprises, it must necessarily happen, that the author's opinions are in many instances identical with those of several other philosophers; and it may appear strange, that we have not, in the foregoing review, noticed any such coincidences. The omission here alluded to has been intentional and premeditated. The author's opinions are his own; and he has founded them on ideas and arguments of so original and peculiar a character, that it is useless to trace the similarity that may exist in a few of his deductions to the notions of some of his predecessors; and it must be just as vain to attempt to support or to oppose reasonings like his by the opinions of any other person, as it would be to quote the authority of Newton or Lacepede for or against the propriety of a given solution of a question in geometry.

There is a peculiarity in the style of this work that is somewhat remarkable: it is a plain, easy simplicity, joined with a strength of manner, that is well adapted for the subject. The author seems to have desired to avoid eliciting the attention of the reader from the matter of his discourse by any alluring graces of manner; yet he sometimes evinces a talent for elegance of composition, that is by no means of an ordinary character. We know nothing more beautifully eloquent than some passages in the third chapter of the first book; one of which, especially, we were much disposed to have transcribed into this article; but the consideration that we had been obliged to pass over in a slight and rapid manner many of the more important parts of the book, prevented its being effected. From the passage here alluded to, and an observation in the Preface, it appears that the author is but thirty-five years of age. If this calculation is correct, his work presents a specimen of early development of the understanding, that must be regarded as one of the most admirable of such instances recorded in history; and this will appear more strikingly evident, when it is considered that the work has been produced under such unfavourable circumstances, as the bodily fatigue and mental distraction attendant on the practice of medicine, to a great extent, and in a provincial city.

---

*Cases of a serious Affection, chiefly occurring after Delivery, Miscarriage, &c. from various Causes of Irritation and Exhaustion; and of a similar Affection unconnected with the Puerperal State.* By MARSHALL HALL, M.D. F.R.S.E. &C. &C. 8vo. pp. 96. Longman and Co. 1820.

THIS work is another specimen of the results of the application of the author's talents for diagnostic pathology, for the purpose of illustrating a particular form of disease; and it confirms the hopes excited by his Treatise on Diagnosis in general, and on that of some of the Disorders of the Digestive System, that he has assumed the cultivation of semiology as the principal object of his exertions. That he possesses eminent qualifications for such a task, is very evident; and this part of pathology has engaged only an inferior, and inappropriate, degree of attention from the generality of modern physicians: those who have studied medicine as a science having made investigations of the causes and identical nature of diseases, without especial reference to those of analogous character, the chief object of their efforts. The ancients, on the contrary, regarded semiological knowledge as that of most importance to the physician, and some of the most precise and valuable indications of this kind in existence are to be found in their writings. Such information was particularly useful in the days of superstition. The prediction of the time at which a disease would terminate in health or in death, was commonly required of them; and the verification of their judgment caused them to be regarded, by the people, as beings possessing faculties which "proxime ad deorum vim natura mortalis possint accedere," as Cicero says of divination. It is from the knowledge they contain of this kind, that we peruse the works of HIPPOCRATES with so much interest; and it forms the most valuable part of the works of ARETÆUS, CŒLIUS AURELIANUS, and ALEXANDER TRALLIANUS. Amongst the productions of the physicians who have lived since the revival of letters that have devoted their attention to this subject, the chief are SYDENHAM, FREIND, DURET (*Hippoc. Coac. Prænot. Comment.*), VALESIUS (*Aph. Hipp. Comment. septem*), PROSPER ALPINUS (*De Præsagiendâ Vitâ et Morte ægrotantium*), FIANUS (*De Signis, &c.*) LOMMIUS (*Med. Observat.*), LEROY (*Du Pronostic dans les Maladies aigues*), PEZOLD (*De Prognosi in Acutis*), COPE (*Demonst. Med. Pract. Prognos.*), HEBERDEN, AVENBRUGGER, PINEL, and especially GRUNER (*Semiotice Physiol. et Pathol. generalem Complexa*), and LAENNEC.

Several authors, in treating of the diseases of the puerperal state, have cautioned practitioners against confounding those disorders dependant on what, to conceal our ignorance, we call irritation, with inflammation of the ordinary character; and many interesting observations on affections of this kind are dispersed in the works of DENMAN; in the paper by Prof. FODERE, inserted a short time since in this Journal; in the writings of GUANI and RUBINI, (the founders of the doctrine which immediately succeeded to Brownism in Italy, of which an exposition was given in the Proëmium to the last volume of this Journal); and some very precise and judicious remarks on them, in a dissertation of FRANCK, entitled *De Venæsectionis apud Puerperas Abusu*; and in that on Puerperal Fever, by the excellent GIANNINI, in the second volume of his work on Fevers in general, (*Della Natura delle Febbri, &c.* Milano, 1809.) But there is nowhere else such an accurate and lucid view of the irritative disorder alluded to as is here displayed by Dr. Hall, in his general abstract, and in his particular illustrations, of the cases which have occurred to his own observation. We agree with him in his assertion, (though it is proper to state that our observations in this respect have been chiefly made in lying-in hospitals,) that "the morbid affection in question constitutes a great proportion among puerperal cases, and a great majority among the fatal ones; and, of these fatal ones, many are daily rendered so by a mistaken use of the lancet;" and we estimate the work before us very highly, as it *must* lessen the frequency of such occurrences in the practice of those who will peruse it, and who have pursued the injurious practice of which the author exposes the impropriety.

This affection appears to arise, the author says, from different sources of irritation and exhaustion, especially as concurring after the fatigue and shock which the system undergoes during labour or abortion.

"The principal source of irritation, is a disordered and loaded state of the alimentary canal; the principal source of exhaustion, uterine hemorrhagy.

"This morbid affection is particularly apt to affect those persons who, previously to delivery, have laboured under a deranged state of the bowels, with constipation, diarrhœa, sickness, &c. It has occurred in females who had, previously to conception, been affected with that complaint which I have, in a little work lately published, denominated the Mimosis Decolor. It has occurred in several persons, who, besides the pallor and icterode complexion of that disorder, had been affected, previously to delivery, with anasarca; and it has occurred in several individuals who had suffered from aphthæ, attended by an irritable state of the stomach and bowels.

"This affection appears to be frequently induced by copious, but especially by protracted, uterine hemorrhagy, the menorrhagia lochialis, imprudent or too copious, or long-continued, lactation, sickness, diarrhœa, &c. It frequently occurs in persons who have, previously to delivery, been reduced by venesection, and other remedies, necessary to subdue an inflammatory disease. It has been induced, or much aggravated, by misapplied venesection after confinement; and it has immediately followed the violent operation of a purge.

"This morbid affection is particularly apt to attack the delicate and feeble in constitution. It is aggravated, or even induced, by too great closeness and warmth of the patient's room, or of the weather. The fatigue of a lingering labour, the violence of labour-pains, anxiety of mind, alarm and hurry, &c. have all appeared to be concurrent causes of this morbid affection.

"It is not impossible that improper articles of diet may also have contributed their share in causing this complaint: but I cannot recal to mind any fact by which this idea is substantiated. This remark must therefore be considered as conjecture only. It is also not improbable, that imprudent fatigue, from too early rising, after delivery, has also had a baneful influence in inducing this malady.

"In some cases the pain attending this affection has been experienced during the latter period of pregnancy. In these instances, is it not probable that the cause exists in the state of the bowels?

"It may here be remarked, that some of the symptoms of the morbid affection in question, which have been continued by protracted uterine hemorrhagy or lactation, have immediately ceased on removing the cause, by the lotion to be recommended hereafter, or by weaning. These causes of some painful affections are too apt to be overlooked in practice: head-ach, palpitation, nervousness, alarm, and sometimes delirium even, have been kept up by these unsuspected causes."

Dissection after death has probably led to erroneous notions respecting the nature of affections of this kind, by developing appearances similar to those which result from the common form of inflammation. Whether they are identical modes of disease or not, is a question of some importance; but the most interesting considerations in regard to therapeutics are these: that the disease above designated often arises in the most debilitated state of the system, and seems to be more readily excited in proportion to the greater degree of weakness, (perhaps because the body is then more susceptible of the influence of irritants,) and that it is increased by the means which lessen inflammation as it ordinarily occurs in a vigorous state of constitution, and is alleviated only by stimulants; and especially by such as are qualified to restore to the vital organs their due energy, which they have lost, in some instances, only because the vitality of the system is concentrated in the uterus or intestines, in consequence of the irritation they have suffered previous to, and during, parturition. It should be considered, too, that medicines of this kind, applied to the stomach for instance, are often the most effectual means of relieving undue action in the uterus, on the principle of derivation, or revulsion, just as a blister removes a pleurisy. They therefore act beneficially in a two-fold manner.

In some cases, the author says, this state of the system has given rise to sudden and unexpected dissolution after parturition; and, occasionally, the patient does not recover from an ill-directed bleeding. Sometimes it terminates fatally, after a more or less urgent or protracted and varied course; at others, there has been long-continued indisposition. It appears principally under the following forms: "1, the acute; 2, the more continued; 3, with general symptoms; 4, with some predominant local affection; 5, as the effect, chiefly, of intestinal irritation; or, 6, of hemorrhagy. The greater number of cases do not, however, admit of being referred to any one of these divisions distinctly or exclusively, but assume a mixed character."

All these forms are illustrated by cases, but we cannot take these into particular consideration: we must confine our analysis to a general abstract, and pass on, therefore, to the section relating to the "Description, Symptoms, &c."

When it has come on in an acute form, the first symptom has occasionally been "severe and long-continued rigor, succeeded by great heat of surface, great frequency of pulse, and some serious affection of the head, or of the abdomen." When the attack is slower and more insidious, "the rigor is less observed, the heat of surface perhaps absent, and there is throbbing pain of the head, with vertigo in the erect posture, or fluttering or palpitation of the heart, or oppressed, hurried, and sighing, breathing, or irritability of the stomach and bowels, &c."

Great intestinal irritation is apt, the author says, to induce a sudden attack, with rigor and much febrile heat, and profuse hemorrhagy will also produce those effects. The general course of the affection, as well as the mode of attack, is considerably influenced by the causes of it, and by the constitution of the patient. The symptoms "refer themselves in general to the head, heart, chest, stomach, bowels, uterus, the muscular system, and to different seats of pain." The author, in his preliminary and general history of this affection, considers the symptoms in each of the seats above mentioned, in succession; and refers to the details of particular cases for the exemplification of them under the several complications in which he has observed them. We shall transcribe at length the general account above alluded to.

"The symptoms which may be referred to the head are the following: severe pain; beating and throbbing; rushing, or cracking noises; vertigo, or turning round of the room, especially on raising the head or assuming the erect position; intolerance of light, and of sound; wakefulness; starting during sleep; awaking hurried and alarmed; with faintness, palpitation, feeling of sinking, of impending dissolution, &c.; being overcome by noise, disturbance, or thinking even; and delirium.

"The heart is, in different cases, affected with palpitation, fluttering, irregular and feeble action; there are beating and throbbing of the carotids, and sometimes even of the abdominal aorta; great rapidity, and sometimes irregularity, of the pulse; faintishness or fainting; urgent demand for the smelling-bottle, fresh air, fanning, bathing of the temples; feeling of impending dissolution; incapability of bearing the erect position; and sometimes early fainting from the use of the lancet.

"The respiration is affected in different cases, with panting, hurry, sighing, great heaving, gasping, blowing, moaning, catching, &c. and, as has been stated, with urgent demand for fresh air. There is sometimes a sense of great and alarming oppression about the chest.

"There is in some cases an irritative cough, in violent fits, or in the form of continual hacking: this cough appears to originate in the larynx or trachea.

"The stomach is liable to become affected with irritability, sickness, retching, vomiting, hiccough, and eructation; the bowels with constipation, or diarrhœa, pain, flatus, distension, &c.

"There are very frequently urgent restlessness, tossing about, and jactitation. In some cases, various spasmodic affections have occurred.

"The seats of pain are usually the head, the side, the iliac region, the loins, the region of the uterus, and the abdomen generally. The pain of the iliac and uterine region, and of the abdomen, is often attended with much tenderness.

"Amongst other symptoms should be mentioned the faintishness, the gasping, the feeling of dissolution, &c. of the patient, which sometimes occur after the first, and even a moderate, bleeding. In some instances the patient has expressed the utmost dread of being bled, from the feeling of aggravation of her suffering, or of dissolution, induced by it."

When treating of the *diagnosis* of this affection, the author states that it is apt, in its various forms, to be mistaken and mistreated for inflammatory diseases of the head, chest, heart, stomach, bowels, uterus, and peritoneum; and especially for puerperal phrenitis and puerperal fever. The distinction is founded, principally, on the presence of some of the more unequivocal symptoms of the subject of this treatise, already described. In the case in which the practitioner has had recourse to blood-letting, the effect of this remedy should be closely remarked: early faintness, increased frequency of the pulse, gasping, internal feeling of dissolution, unremitting pain, &c. are circumstances which ought, at least, to lead to the greatest caution and circumspection with regard to the further use of this remedy. In all cases, the author adds, the colon and rectum should be unloaded by glysters. This measure affords a source of diagnosis of the utmost importance, and in the opportunity it gives for the observation of the state of the intestinal contents.

The treatment the author inculcates consists in removing the causes, whether of irritation or exhaustion, and in obviating the effects already induced by them. Intestinal irritation, as already indicated, is considered as the most frequent cause; and the measures for the removal of this "must be at once mild and efficient; otherwise, exhaustion, on the one hand, and irritation only partially removed, on the other, may prove the source of the greatest danger. It is equally essential to give nourishment, and to avoid loading or disordering the stomach; and the benefit of the wisest plan may be counteracted, by imprudent exposure to fatigue, exertion, hurry, agitation, or anxiety." The author next proceeds to illustrate these principles.

"Intestinal irritation must be removed by aperient medicines and by enemata.

"With regard to the former, small doses of calomel, and draughts with rhubarb and sulphat of magnesia, have appeared to me to be the best. One point is of the greatest importance,—it is the union, with the purgative, of a proper dose of opium, or of a stimulant medicine; and a point of little inferior importance, is the administration, before, during, and after, the action of the purgative medicine, of proper nourishment. With the calomel, I have given a small quantity of opium; and with the rhubarb and sulphat of magnesia, a little of the tinctura cardamomi comp.

"It is impossible to say too much in recommending the use of enemata. By their means the intestine is unloaded effectually, and without the exhaustion experienced from the action of efficient purgative medicine, and without disordering the stomach. It is, of course, needless to represent the importance of an exact inspection of the effect of enemata and of the purgative medicine administered.

"It is, in the next place, necessary to notice the means for obviating the various sources of exhaustion. If this be uterine hæmorrhagy, the following application is, I think, most effectual in arresting it: A lotion is prepared by dissolving from one to two drachms and a half of sulphat of zinc in a pint of soft water: a scroll of linen is then made of a proper form and bulk to fill the vagina; this scroll is then fully imbued with the lotion, introduced into the vagina, and renewed frequently. The same lotion may also be applied externally.

"The other remedies which I have found useful, are the tinctura opii, the tinctura camphoræ comp., the sp. ammoniæ aromat., æther, wine, and similar remedies: in one instance, opium, the ext. hyoscyami, and the carbonas ammoniæ, were combined, with the best effect.

A proper combination of these remedies induces quiet sleep, prevents the exhaustion which would otherwise ensue from the administration of the purgative, and relieves many of the distressing symptoms of this alarming complaint.

"Similar objects are obtained by the due administration of nourishment: this should consist of chicken-broth, one part of milk and two or three of water thickened with arrow-root, &c. Something of this kind should be given, in small quantities, every hour or oftener, and especially as stated."

Severe local pain is to be alleviated by various auxiliaries. When the head is thus affected, a cold lotion may be applied to it, and warm fomentation to the feet. Sometimes leeches to the temples may be advisable; but the author considers, that, when the head is affected from exhaustion, even leeches might appear improper. Venesection, he says, must always be a very hazardous remedy, and ought, he thinks, to be proscribed in these cases altogether. The same remarks are applicable to other cases of local pain. The French practitioners often use what they call *flying blisters* as revulsives in such cases, and we think with beneficial effects, when any important organ is the seat of much pain. The flying blisters are common blistering-plasters applied only for two or three hours, generally to the inside of the thighs or to the calves of the legs: a little redness of the skin is thus produced, without vesication, which might itself be a serious cause of exhaustion to a person already much debilitated.

In the attack of jactitation, Dr. Hall recommends a draught of laudanum and aromatic spirit of ammonia, free ventilation, &c. This part of the work concludes with some remarks on the importance of quietude and of good domestic management in cases where the occurrence of the affection under consideration may be feared, or has already taken place. One remark, in particular, amongst those last alluded to, strikes us in regard to its importance.

"In those cases in which the sleep is disturbed, and the moment of awaking is attended by great alarm and agitation, the sleep should be watched: if there be any agitation from dreaming or otherwise, the patient should be gently and cautiously awakened, and soothed and calmed if there should be alarm; and the best mode of awaking has appeared to me to be by offering a little nourishment, the mind by this means being immediately collected to understand the state of things."

A state somewhat similar to that above considered may arise after abortion, or independantly of the puerperal state; and in men, from various analogous causes; and requires similar modes of treatment. This view of it was more particularly considered, as arising from intestinal disorder, in the author's essay on the "Mimoses."

The cases which are related in this work form a highly valuable part of it. The descriptions of the malady are given in them in very strong traits, and mark a rare talent for clinical observation. The deleterious effects of blood-letting are forcibly shown, and well contrasted with the treatment the author advises. The infatuation with which some of the practitioners who attended previously to Dr. Hall had used the lancet in some of them, is lamentable; and it makes us particularly anxious to press on the attention of our readers this excellent addition to clinical medical literature.

### CRITICAL ANALYSIS

OF

